# Quantifying the economic gains associated with COVID-19 vaccination in the Canadian population: A cost-benefit analysis

**DOI:** 10.14745/ccdr.v49i06a03

**Published:** 2023-06-01

**Authors:** Ashleigh R Tuite, Victoria Ng, Raphael Ximenes, Alan Diener, Ellen Rafferty, Nicholas H Ogden, Matthew Tunis

**Affiliations:** 1 Centre for Immunization and Respiratory Infectious Diseases, Public Health Agency of Canada, Ottawa, ON; 2 Dalla Lana School of Public Health, University of Toronto, Toronto, ON; 3 Public Health Risk Sciences Division, National Microbiology Laboratory, Public Health Agency of Canada, Saint-Hyacinthe, QC and Guelph, ON; 4 Policy Research, Economics, and Analytics Unit, Strategic Policy Branch, Health Canada, Ottawa, ON; 5 Institute of Health Economics, Edmonton, AB

**Keywords:** SARS-CoV-2, COVID-19, vaccination, cost-benefit analysis, health economics, modelling

## Abstract

**Background:**

Vaccination has been a key part of Canada’s coronavirus disease 2019 (COVID-19) pandemic response. Although the clinical benefits of vaccination are clear, an understanding of the population-level benefits of vaccination relative to the programmatic costs is of value. The objective of this article is to quantify the economic impact of COVID-19 vaccination in the Canadian population between December 2020 and March 2022.

**Methods:**

We conducted a model-based cost-benefit analysis of Canada’s COVID-19 vaccination program. We used an epidemiological model to estimate the number of COVID-19 symptomatic cases, hospitalizations, post-COVID condition (PCC) cases, and deaths in the presence and absence of vaccination. Median, lower and upper 95% credible interval (95% CrI) outcome values from 100 model simulations were used to estimate the direct and indirect costs of illness, including the value of health. We used a societal perspective and a 1.5% discount rate.

**Results:**

We estimated that the costs of the vaccination program were far outweighed by the savings associated with averted infections and associated downstream consequences. Vaccination increased the net benefit by CAD $298.1 billion (95% CrI: 27.2–494.6) compared to the no vaccination counterfactual. The largest benefits were due to averted premature mortality, resulting in an estimated $222.0 billion (95% CrI: 31.2–379.0) benefit.

**Conclusion:**

Our model-based economic evaluation provides a retrospective assessment of COVID-19 vaccination during the first 16 months of the program in Canada and suggests that it was welfare-improving, considering the decreased hospitalizations and use of healthcare resources, deaths averted and lower morbidity from conditions such as PCC.

## Introduction

The availability of coronavirus disease 2019 (COVID-19) vaccines marked a turning point in Canada’s pandemic response, allowing for a reduced reliance on non-pharmaceutical interventions (NPIs) to protect population health. Despite the demonstrated effectiveness of COVID-19 vaccines for preventing severe outcomes associated with severe acute respiratory syndrome coronavirus 2 (SARS-CoV-2) infections ((1)), quantifying the effect of COVID-19 vaccination programs on Canada’s pandemic trajectory is challenging. Mathematical modelling can be used to compare the Canadian pandemic experience to a counterfactual scenario of how the pandemic might have unfolded in the absence of vaccination. The modelling has shown the substantial clinical benefits of COVID-19 vaccination for preventing SARS-CoV-2 infections, hospitalizations and deaths ((2,3)).

Although the population impacts of COVID-19 vaccination are frequently discussed in terms of health outcomes, undertaking a cost-benefit analysis allows for a more comprehensive evaluation. In a cost-benefit analysis, all outcomes are valued in monetary terms allowing for the inclusion of non-health outcomes ((4)). This lens allows for a more complete accounting of the costs of illness, including reduced quality-of-life and labour market effects due to illness-associated disability and mortality, in addition to the direct healthcare costs ((5)). This is particularly relevant for post-COVID condition (PCC; also known as long COVID), given emerging data showing the high prevalence of PCC in countries experiencing high rates of SARS-CoV-2 infection ((6,7)). Additionally, there is measurable negative impact of PCC on workforce productivity, including worker absenteeism and exit from the workforce ((8,9)).

Initial economic evaluations of COVID-19 vaccination in North America have demonstrated that COVID-19 vaccination programs have resulted in substantial economic benefit ((10,11)). An analysis of Canada’s vaccination program estimated a net cost-benefit of −$0.4 billion to $2.1 billion when considering treatment costs and lost productivity due to illness, and a further $27.6 billion benefit due to prevented mortality ((11)). Notably, this study used a statistical model that did not account for the transmissibility of SARS-CoV-2, such that the estimates of COVID-19 cases averted with vaccination are likely to be underestimated.

We used transmission modelling to retrospectively quantify the economic impact of vaccination in the Canadian population due to the prevention of SARS-CoV-2 infections, and associated hospitalizations, deaths and PCC cases. The analysis focuses on a 16-month period, following the first authorization of vaccines in December 2020 until March 2022. Over this time period, approximately 87.5% of Canadians aged five years and older had received at least one vaccine dose, 84% had completed their primary series and 48.8% had received three or more doses ((12)).

## Methods

We conducted a cost-benefit analysis of COVID-19 vaccination in the Canadian population. We used an epidemiological model of SARS-CoV-2 transmission to assess the impact of vaccination on COVID-19 burden and evaluate the net benefit associated with vaccination.

### Transmission model overview and scenarios

We adapted a previously reported age-structured agent-based model that describes the transmission of SARS-CoV-2 in the Canadian population to estimate COVID-19 cases in the presence and absence of COVID-19 vaccines ((3,13)). The model simulates transmission in a general community setting and excludes outbreaks in discrete settings such long-term care homes, which experienced high rates of infection. Model outputs were validated by comparison with available administrative data ((3)).

We developed two alternative scenarios using model parameters that were otherwise unchanged from the previously described analysis ((3)): a “what happened” baseline scenario and a “no vaccine” counterfactual scenario. The baseline scenario reflected the observed rollout of vaccination programs, in terms of age groups eligible for vaccination and coverage achieved ((14)). The model time period included the emergence of the Omicron variant of concern, which triggered an expedited rollout of third doses in the general population in the winter of 2021/2022 ((15)); additional details about the modelled time period are provided in Ogden *et al.* ((3)). The baseline included observed levels of NPIs over this period since the availability of vaccines did not result in the immediate removal of NPIs.

The counterfactual scenario represented what might have occurred in the absence of vaccination, with continued NPI use to mitigate recurring waves of infection and health system strain. The timing of introduction and lifting of NPIs (“shutdowns”) in the counterfactual scenario was based on intensive care unit (ICU) occupancy, with thresholds based on observed ICU occupancy when NPIs were introduced in the second wave of the pandemic (September 2020 to February 2021). Because the model is stochastic, timing and duration of NPI use varied across model runs for the counterfactual scenario. In both scenarios, lifting of NPIs occurred gradually over a four-week period.

The model population size was 100,000 and outputs were rescaled to represent the size of the Canadian population. Each model scenario was run 100 times. The model was run from February 7, 2020, to March 31, 2022, and outcomes were calculated from December 14, 2020, onwards, to capture the period of divergence between the baseline and counterfactual scenarios following the start of vaccination. Model outputs included COVID-19 clinical cases (all cases experiencing symptoms, regardless of severity), hospitalizations, ICU admissions and deaths in the baseline scenario compared to the counterfactual scenario. We also calculated the number of vaccine doses administered for the baseline scenario, and number and duration of shutdowns. We used the median, lower 95% credible interval (CrI) and upper 95% CrI output values for the economic analysis.

### Estimation of post-COVID condition cases averted

Model-projected clinical cases (excluding fatal cases) for the two scenarios were used to estimate the incidence of PCC following SARS-CoV-2 infection in the presence and absence of vaccination. Where possible, we used the World Health Organization case definition of PCC ((16)). The probability of developing PCC among clinical cases was derived from a general population cohort with age and sex-matched controls ((17)). We did not apply differential risks of developing PCC by age or infection severity.

Vaccination was assumed to prevent PCC two ways: first, by preventing SARS-CoV-2 infection; and second, by reducing the likelihood of developing PCC if infected. Vaccine effectiveness for preventing infection was assumed to be dependent on the predominant circulating variant of concern at the time of infection ((3)), while vaccine effectiveness for preventing PCC following infection was assumed to be constant (15%), regardless of the infecting variant of concern ((18)). Protection against PCC was only assumed among people who had received two or more vaccine doses prior to infection. We did not model a reduction in PCC risk among people vaccinated after SARS-CoV-2 infection and did not include waning of protection from PCC over the model time horizon.

### Economic impact of COVID-19 cases averted

We estimated the total costs of illness, including direct and indirect costs and the value of health (morbidity and mortality) ((5)) to enumerate the economic impact of COVID-19 cases averted due to vaccination from a societal perspective. We used a lifetime time horizon to enumerate the costs and health consequences associated with COVID-19-attributable mortality. For PCC, we estimated costs and health effects for the first year following onset, given limited data on the longer-term trajectory of PCC. Costs are in 2021 Canadian dollars and where necessary were converted using the Canadian Consumer Price Index ((19)). We used a discount rate of 1.5% per year. Input parameters for the economic model were derived from the published studies, wherever possible, and by assumption and expert opinion otherwise (**Table 1**).

**Table 1:** Input parameters for the economic model

Applicable outcome	Parameter	Value	Source
Direct costs			
Clinical case	Net medical cost per outpatient case ($)	165.2	Tsui *et al.* ((20))
	PCR test ($)	60.7	Campbell *et al.* ((21))
Hospitalization (including ICU)	Healthcare cost per hospitalization ($)	25,103	CIHI ((22))
PCC case	Cost per case ($, in first year)	9,683	Institute for Health Economics, *personal communication*
Vaccination	Vaccine cost per dose ($)	30	Office of the Auditor General of Canada ((23))
	Administration costs per dose ($)	34	Office of the Auditor General of Ontario ((24))
	Other programmatic costs per dose ($)	27	Assumption based on Sah *et al.* ((10))
Indirect costs			
All	Average employment income, age 16 years and older ($)	49,095	Statistics Canada ((25))
	Average employment income, ages 25–54 years ($)	58,811	Statistics Canada ((25))
	Average employment income ($)	Age-specific values	Statistics Canada ((25))
Productivity loss			
Clinical case	Time off work (days)	10	Government of Canada ((26))
Hospitalization (including ICU)	Length of stay in hospital (days)	13	CIHI ((22))
	Time from hospital discharge to return to work (days)	27	Chopra *et al.* ((27))
PCC case	Proportion of PCC cases with ongoing symptoms at one year	0.15	Waters and Wernham ((8))
	Average reduction in earning during first six months of illness (%)	11	Wulf Hanson ((28))
	Average annual reduction in salary (%)	8.3	Extrapolated from ((8)) and ((28))
Vaccination	Time off work to receive vaccine (days)	0.4	Government of Alberta ((29))
	Proportion unable to work one day post-vaccination, dose 1	0.05	Rosenblum *et al.* ((30))
	Proportion unable to work one day post-vaccination, dose 2	0.23	Rosenblum *et al.* ((30))
	Proportion unable to work one day post-vaccination, booster doses	0.23	Assumption
All	Labour force participation, age 15 years and older (%)	64.6	Statistics Canada ((31))
	Labour force participation, ages 25–54 years (%)	87.0	Statistics Canada ((31))
	Labour force participation (%)	Age-specific values	Statistics Canada ((31))
QALY loss			
Clinical case	0–14 years	0.0050	Kirwin *et al.* ((32))
	15–64 years	0.0077	Kirwin *et al.* ((32))
	65 years and older	0.012	Kirwin *et al.* ((32))
Hospitalization (including ICU)	QALY loss (per year)	0.58	Kirwin *et al.* ((32)); adjusted for length of hospital stay
	QALY loss on discharge (per case)	0.1	Kirwin *et al.* ((32))
PCC case	QALY loss (1 year following discharge)	0.2937	Weighted decrement for common chronic conditions associated with PCC, Institute for Health Economics, *personal communication*
Death (net present value)	0–9 years	41.37	Kirwin *et al.* ((32))
	10–19 years	37.19	Kirwin *et al.* ((32))
	20–29 years	33.37	Kirwin *et al.* ((32))
	30–39 years	29.4	Kirwin *et al.* ((32))
	40–49 years	24.9	Kirwin *et al.* ((32))
	50–59 years	20.18	Kirwin *et al.* ((32))
	60–69 years	15.36	Kirwin *et al.* ((32))
	70–74 years	10.35	Kirwin *et al.* ((32))
	75 years	5.17	Kirwin *et al.* ((32))
Vaccination	QALY loss if experience adverse event following immunization	0.00027	Sandmann *et al.* ((33))
Other			
PCC case	Vaccine effectiveness for preventing PCC following infection	0.15	Al-Aly ((18))
Clinical case	Percent of clinical cases developing PCC	12.7 (7.8–17.0)	Ballering ((17)); Thompson ((34))
All	Vaccine wastage (%)	3	Office of the Auditor General of Ontario ((24)); for the period of December 2020 to January 2022
	Percent of cases tested by PCR	20	Statistics Canada ((35)) and assumption
	Discount rate (%)	1.5	CADTH ((36))
	Cost per QALY threshold ($)	30,000 (20,000–100,000)	Ochalek *et al.* ((37))

Abbreviations: CADTH, Canadian Agency for Drugs and Technologies in Health; CIHI, Canadian Institute for Health Information; ICU, intensive care unit; PCC, post-COVID condition; PCR, polymerase chain reaction; QALY, quality-adjusted life years

Direct costs included medical costs due to COVID-19 cases, comprising outpatient care and hospitalization for acute COVID-19 and treatment of PCC. Vaccination program costs encompassed the cost of purchasing and administering COVID-19 vaccines, including estimated wastage, as well costs associated with delivery of the program to the population, such as storage and transportation, clinic set up, and advertisement and outreach ((10)). The cost of wasted doses excluded vaccine administration costs.

Indirect costs included the value of lost production due to days of employment loss due to illness, disability, death or caregiving responsibilities, as well as production losses associated with time to receive a vaccine and possible adverse events following immunization (AEFI). We did not include out-of-pocket medical costs (e.g. pharmaceutical costs). Productivity loss was quantified using the human capital approach ((4)). We used age-specific estimates of labour force participation for the years 2020 and 2021 ((31)) and average employment income for 2020 ((25)). Caregiver costs were based on estimates of the average employment income and labour force participation of people aged 25–54 years, adjusted for estimated caregiver productivity loss ((38)). We included caregiver costs associated with outpatient infections in children less than 15 years of age, and caregiver costs for hospitalized cases for those age younger than 15 years and 65 years and older. To estimate production loss associated with receiving the vaccine, we used labour force participation rates and average salary in the population aged 16 and older to account for caregiver time off work to accompany children to vaccination appointments.

Health impacts included disutility from symptomatic infection, hospitalization, PCC, death and AEFI. Quality-adjusted life years (QALYs) were monetized using a cost per QALY threshold of $30,000 ((37)). Net benefit was estimated using the “no vaccination” counterfactual scenario as the baseline. The transmission model was constructed in AnyLogic 8 Professional 8.7.2 and the economic analysis was conducted using R ((39)).

### Sensitivity analyses

To address uncertainty around vaccine costs, including programmatic costs, we estimated a threshold cost to determine the maximum vaccine cost per dose for which a COVID-19 vaccination would have been cost beneficial. We assumed that administration costs were fixed at the value used in the main analysis.

We explored cost per QALY thresholds values of $20,000, $50,000 and $100,000 in sensitivity analysis. We assessed lower (7.8%) and higher (17.0%) estimates of risk of PCC ((34)) to evaluate how these estimates impacted findings.

We re-estimated production losses using the friction cost approach. In contrast with the human capital approach, the friction cost approach assumes that after a “friction period”, workers who have left the workforce will eventually be replaced by currently unemployed workers ((40)). We used a three-month friction period for people with PCC or who died of COVID-19 ((41)).

## Results

With vaccination, the average Canadian population experience of the pandemic from December 2020 to March 2022 was represented in the model as a total of three shutdown periods for a total duration of 112 days. In contrast, in the absence of vaccination but with continued implementation of NPIs in the face of healthcare system strain, we would have expected four extended shutdown periods (95% CrI: 3–5) for a total duration of 343 days (95% CrI: 268–399).

**Figure 1:**
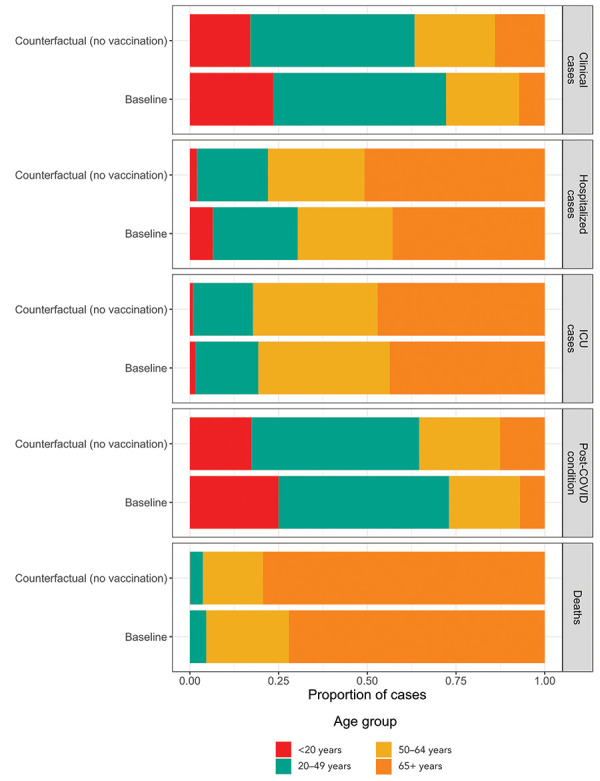
Age distribution of COVID-19 health outcomes for the two model scenarios^a,b^ Abbreviations: COVID-19, coronavirus disease 2019; ICU, intensive care unit ^a^ The proportions of cases by age group are shown for the baseline and no vaccination counterfactual scenarios, based on median model estimates ^b^ The total number of health outcomes for the two scenarios are provided in Table 2

Model-estimated health outcomes used for the economic analysis are presented in **Table 2** and **Figure 1**. For the median and upper bound model estimates, incidence of all COVID-19 outcomes was higher in the “no vaccine” counterfactual scenario compared to the baseline. For the lower bound model estimates, although the incidence of symptomatic infections and PCC was higher in the baseline scenario, the occurrence of hospitalizations and deaths was higher for the “no vaccination” counterfactual, due to the effectiveness of vaccination for preventing severe outcomes.

**Table 2:** Model-projected health outcomes and outcomes averted^a^ in the Canadian population, December 14, 2020 to March 31, 2022

Health outcome	Scenario		Averted (counterfactual minus baseline)
	Baseline	Counterfactual (no vaccination)^b^	
Clinical cases	13,618,980 (11,709,360–15,704,590)	24,713,530 (10,327,700–30,926,840)	11,094,550 (−1,381,660–15,222,240)
Hospitalized cases (excluding ICU)	86,090 (50,930–131,510)	1,270,100 (296,480–1,880,730)	1,184,010 (245,540–1,749,220)
ICU cases	25,660 (14,440–41,810)	375,730 (86,660–590,300)	350,070 (72,220–548,480)
PCC cases	1,566,540 (1,341,560–1,815,660)	3,070,700 (1,301,046–3,823,630)	1,504,160 (−40,510–2,007,970)
Deaths	10,640 (4,180–19,770)	534,800 (83,240–819,500)	524,160 (79,060–799,730)

Abbreviations: COVID-19, coronavirus disease 2019; ICU, intensive care unit; PCC, post-COVID condition

^a^ Median, lower and upper bounds

^b^ For the counterfactual scenario, introduction of non-pharmaceutical interventions (NPIs) occurred when COVID-19 ICU occupancy exceeded three cases per 100,000 population and were implemented for an initial period of six weeks. After six weeks, NPIs were lifted if ICU occupancy was less than or equal to one per 100,000. If occupancy exceeded the one per 100,000 threshold after six weeks, NPIs were extended by four-week intervals until occupancy no longer exceeds one per 100,000. Lifting of NPIs occurred over a four-week period, with gradual removal of closures and physical distancing until reaching a return to pre-COVID-19 contact rates

Vaccination was associated with 6.61 million (95% CrI: 0.88–10.8) QALYs gained and increased the net benefit by $298.1 billion (95% CrI: 27.2–494.6) compared to the “no vaccination” counterfactual (**Table 3**). This represents a benefit-cost ratio of 26.7 (3.6–43.3). The largest benefits were due to averted premature mortality, resulting in an estimated $222.0 billion (95% CrI: 31.2–379.0) benefit.

**Table 3:** Net benefit of vaccination relative to the “no vaccination” counterfactual scenario, by health outcome and cost component

Health outcome	Incremental benefits ($ billions)		
	Direct	Indirect	Total
Clinical cases	2 (−0.176–2.77)	12.8 (−1.1–18)	14.8 (−1.28–20.8)
Hospitalized cases (including ICU)	29.6 (6.26–45.9)	10.9 (2.31–17)	40.6 (8.57–62.9)
PCC cases	14.8 (0.0342–19.8)	17.5 (0.222–23.7)	32.3 (0.256–43.5)
Deaths	N/A	222 (31.2–379)	222 (31.2–379)
Vaccination	−7.56 (−7.54–−7.59)	−4.05 (−4.04–−4.07)	−11.6 (−11.6–−11.7)
Total	38.83 (−1.426–60.88)	259.3 (28.59–433.7)	298.1 (27.16–494.6)

Abbreviations: COVID-19, coronavirus disease 2019; ICU, intensive care unit; N/A, not applicable; PCC, post-COVID condition

We estimated that if the costs of vaccination were 64 times (95% CrI: 7–104) the assumed baseline value, the vaccination program would still have provided a net benefit, when using a societal perspective that includes both direct and indirect costs. For the lower bound model estimate, this means that for a cost of up to $410 per dose (excluding administration costs), the vaccination program would be considered cost-beneficial; for the median and upper bound estimates, these values are $3,630 and $5,950 per dose, respectively. Considering direct medical costs and monetized QALYs only, which reflects the healthcare payer perspective that is typically used in healthcare decision-making, a cost per dose of up to $390, $2,910 and $4,640 would be cost beneficial for the lower, median and upper bound scenarios, respectively.

**Figure 2:**
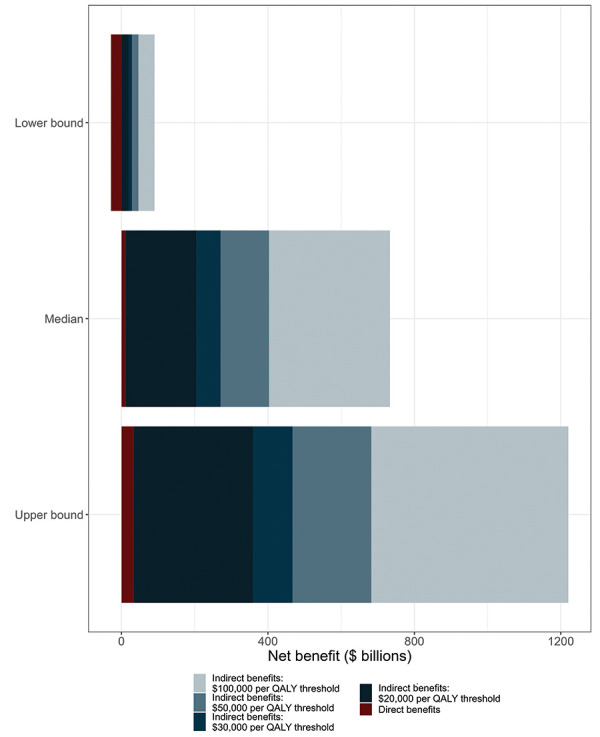
Net benefit associated with Canada’s COVID-19 vaccination program, for different cost per quality-adjusted life year thresholds, December 14, 2020–March 31, 2022^a,b^ Abbreviations: COVID-19, coronavirus disease 2019; QALY, quality-adjusted life years ^a^ Results are shown for lower bound, median, and upper bound model-based estimates of health outcomes averted by vaccination compared to a “no vaccination” counterfactual scenario ^b^ QALYs were converted to monetary values by multiplying QALYs gained by the cost per QALY threshold. The main analysis used a threshold of $30,000 per QALY

The use of higher cost per QALY thresholds increased the welfare gain of vaccination compared to the counterfactual (**Figure 2**), with a maximum benefit of $1.25 trillion for the upper bound model estimate and a threshold of $100,000 per QALY. Using a lower threshold of $20,000 per QALY and the most conservative model estimates of vaccine impact resulted in an estimated net benefit of $18.3 billion.

Lower or higher risk of PCC following infection did not have a substantial impact on estimated benefit of the vaccination program. The net benefit was estimated as $285.7 billion (95% CrI: 27.1–477.8) and $309.1 billion (95% CrI: 27.2–509.3), when PCC occurred in 7.8% or 17% of clinical cases, respectively.

The net benefit of vaccination was reduced when using the friction cost instead of the human capital approach to estimate production losses but remained large at $251.0 billion (95% CrI: 21.6–406.3). Most of the reduced benefit was due to lower estimated indirect costs due to mortality, a reduction of $44.4 billon (95% CrI: 5.4–84.5).

## Discussion

We estimate that Canada’s COVID-19 vaccination program resulted in tens to hundreds of billions of dollars in monetary benefit compared to a situation without vaccination and exclusive reliance on NPIs to control transmission. The costs of the vaccination program were far outweighed by the savings associated with averted infections and associated downstream consequences. Although the largest benefit was derived from averted premature mortality, the indirect benefit associated with reduced illness and disability was also substantial.

Our findings are consistent with an analysis of New York City’s COVID-19 vaccination campaign ((10)). Despite different epidemiological methods and a different healthcare system, that study also demonstrated substantial cost savings associated with the city’s COVID-19 vaccination program ((10)). A recent analysis of Canada’s vaccination program also found the vaccination program to be cost-beneficial, with a net monetary benefit of −$0.4 billion to $2.1 billion, with an additional $27.6 billion in economic benefit associated with averted mortality ((11)); this analysis, which did not use a transmission model to estimate health outcomes averted with vaccination, likely underestimated the benefits of the program. For comparison, the 2022 analysis ((11)) estimated that the vaccination program prevented 30,900 deaths from January 2021 to May 2022, while our analysis estimated 524,000 deaths averted over a similar period (December 2020 to March 2022). Another model-based analysis ((2)) estimated 314,100 deaths averted in the first year of Canada’s vaccination program (December 2020 to December 2021).

### Strengths and limitations

Our estimates of benefit do not include a full accounting of the societal impact of the vaccines for speeding economic recovery ((42)). The counterfactual model showed that without vaccination, the number of days with NPIs in place could have been three times as high as what was observed. A recent analysis estimated that a six-month delay in access to vaccines would have resulted losses of $156 billion in economic activity (or 12.5% of Canada’s gross domestic product) ((11)). Relatedly, we did not include the societal costs associated with the prolonged use of NPIs or the downstream effects on the healthcare system resulting from with a higher burden of COVID-19 cases and deferral of care for other health needs ((43,44)); the inclusion of these costs would further increase the economic benefit associated with vaccination.

Due to the confidentiality of COVID-19 vaccine pricing information, we did not use or have access to these data. Instead, we used publicly available estimates of the average cost of the vaccine per dose, which may over or under-estimate the actual cost of vaccines. Similarly, information about other costs associated with the vaccination programs, including storage, transportation, outreach and wastage, were based on public information, assumption and expert opinion. Despite uncertainty in these values, we estimated that the costs of vaccination could have been 10 to 100-fold greater and still been considered a cost-beneficial intervention.

We compared the observed pandemic trajectory to a “no vaccination” counterfactual scenario where implementation of NPIs was tied to ICU capacity. The precise nature of how the pandemic might have been managed in Canada had vaccines not become available is unknowable. Notably, we did not model interventions such as continued used of masking or improvements in ventilation, which might have been more widely adopted had vaccination not become available. Given the uncertainty associated with the counterfactual, we included lower and upper bound model outputs in the economic evaluation. We also noted that vaccination remained a cost-beneficial intervention for the conservative lower bound estimate, where the model predicted higher numbers of symptomatic cases with vaccination, but reduced severe infections, compared to the counterfactual.

The benefit of vaccination for prevention of PCC remains challenging to quantify. We limited our estimates of PCC impacts in the first year following infection, given uncertainty about the longer-term trajectory of illness among cases and thus likely underestimated the total burden associated with PCC. Our model-derived estimates of PCC in the Canadian population of 4.1% (range: 3.5%–4.7%) over the modelled time period are aligned with Canadian survey data indicating that 4.6% of the Canadian population aged 18 years and older reported ongoing symptoms at least three months after SARS-CoV-2 infection, based on data collected between April and August 2022 ((35)). We assumed that the risk of developing PCC applied equally, regardless of severity of initial infection. The data suggested an increased risk of PCC among more severe cases ((7,35)) and therefore, the estimated impact of vaccination for preventing PCC may be underestimated in our model. Sensitivity analyses revealed that different assumptions about the rate of PCC are unlikely to be very influential on the costs averted by the vaccination program.

We monetized QALYs to estimate the benefits associated with averted COVID-19 morbidity and mortality. The value of statistical life (VSL) approach is an alternative for quantifying the health impacts of an intervention in cost-benefit analyses ((4)). The VSL allows for an accounting of the impact of reductions in mortality risk on all aspects of well-being, such as averted medical expenses and the pain and suffering associated with illness ((4)). It has the disadvantage of typically not accounting for the morbidity associated with non-fatal cases ((4)). A comparison of VSL and monetized QALY approaches for human papillomavirus vaccination programs showed that VSL was associated with higher estimated benefit ((4)). Given the large burden of morbidity associated with COVID-19, we used a monetized QALY approach but note that alternate approaches may result in different estimates of the monetary benefit of COVID-19 vaccination.

## Conclusion

Our model-based economic evaluation provides a retrospective assessment of COVID-19 vaccination during the first 16 months of the program in Canada and suggests that it was welfare-improving, considering decreased hospitalizations and use of healthcare resources, deaths averted and lower morbidity from conditions such as PCC. Including the benefits associated with the economic recovery through fewer days in shutdown scenarios would show even greater increases in net benefits. This analysis may help build a foundation for assessment of cost effectiveness and vaccine procurement decisions in future pandemics.
